# NVCL-Based Hydrogels and Composites for Biomedical Applications: Progress in the Last Ten Years

**DOI:** 10.3390/ijms23094722

**Published:** 2022-04-25

**Authors:** Alejandra Gonzalez-Urias, Angel Licea-Claverie, J. Adriana Sañudo-Barajas, Mirian A. González-Ayón

**Affiliations:** 1 Centro de Graduados e Investigación en Química, Instituto Tecnológico de Tijuana, Tecnológico Nacional de México, Apartado Postal 1166, Tijuana 22454, Mexico; maria.gonzalezu@tectijuana.edu.mx (A.G.-U.); aliceac@tectijuana.mx (A.L.-C.); 2 Centro de Investigación en Alimentación y Desarrollo, A. C. Carretera a Eldorado Km 5.5, Culiacán 80110, Mexico

**Keywords:** poly(*N*-vinylcaprolactam), hydrogels, composites, chemical cross-linked, biomedical applications

## Abstract

Hydrogels consist of three-dimensionally crosslinked polymeric chains, are hydrophilic, have the ability to absorb other molecules in their structure and are relatively easy to obtain. However, in order to improve some of their properties, usually mechanical, or to provide them with some physical, chemical or biological characteristics, hydrogels have been synthesized combined with other synthetic or natural polymers, filled with inorganic nanoparticles, metals, and even polymeric nanoparticles, giving rise to composite hydrogels. In general, different types of hydrogels have been synthesized; however, in this review, we refer to those obtained from the thermosensitive polymer poly(*N*-vinylcaprolactam) (PNVCL) and we focus on the definition, properties, synthesis techniques, nanomaterials used as fillers in composites and mainly applications of PNVCL-based hydrogels in the biomedical area. This type of material has great potential in biomedical applications such as drug delivery systems, tissue engineering, as antimicrobials and in diagnostic and bioimaging.

## 1. Introduction

Hydrogels are defined as three-dimensional cross-linked polymer networks with the ability to uptake a lot of water or biological fluids and hold a large amount of them [1], more than 400 times its original weight, and more than 20% of their dry weight [2]. These materials can be made by physical or chemical bonds [3]. The latter are characterized by being highly stable materials with viscoelastic properties, which can be swelling in aqueous medium or being a good solvent, without dissolving. The three-dimensional network structure can be formed by cross-linking polymer chains through covalent or hydrogen bonding, van der Waals forces or physical entanglements [4]. These polymer networks have a wide range of industrial applications such as oil recovery, pharmaceutical, agriculture, textile, and water treatment [2]. By introducing different functional groups into the structure, hydrogels can respond to external environmental stimuli such as temperature, pH, light, electric and magnetic stimuli, and salts, changing their size and shape, known as smart or stimuli-responsive hydrogels [5].

To obtain materials with improved properties, hydrogels from polymers of natural and synthetic origin, and combinations of both, have been developed. Gelatin, alginate, chitosan, cellulose, dextran, fibrin, starch, hyaluronic acid, collagen, silk, and agarose are examples of natural polymers used to obtain hydrogels. On the other hand, poly(ethylene glycol), poly(hydroxyethyl methacrylate), poly(acryl amide), poly(vinyl alcohol), poly(*N*-isopropylacrylamide), and poly(*N*-vinylpyrrolidone) are examples of synthetic polymers that have been used to obtain fully synthetic or semi-synthetic polymeric networks [6]. All of them have applications such as contact lenses, wound dressings, drug delivery systems (via peroral, rectal, vaginal, ocular, transdermal implants), tissue engineering or as hygiene products [7]. In particular, temperature-responsive hydrogels have been extensively studied for different applications in biomedicine, since they can undergo a volume phase transition at a specific temperature, showing a change from a swollen to shrunken state [8]. Poly(*N*-isopropylacrylamide) is the most popular temperature-responsive polymer. Nevertheless, it has been reported that its degradation products originate toxic compounds, which limits its application in the biomedical area [9]. Otherwise, poly(*N*-vinylcaprolactam) (PNVCL) is biocompatible and the second most popular thermoresponsive polymer, studied since 1968 [10]. However, the first covalently linked hydrogel from PNVCL was reported in 1996 [11], and the first reports were physical gels. Different techniques have been used to obtain chemical cross-linking hydrogels such as free-radical cross-linking polymerization [12,13], photopolymerization [14], radiation polymerization [15,16,17], frontal polymerization [18,19,20], sequential polymerization [21], and sol–gel technology [22]. However, usually hydrogels elaborated from purely synthetic or natural polymers have poor mechanical properties [23]. Therefore, hydrogels or composite hydrogels with more than one constituent have been developed to provide new physical, chemical or biological properties [24]. In this sense, metal and metal oxide nanoparticles [25,26,27], carbon-based nanomaterials [28,29,30,31,32] and polymeric nanofillers [33,34] have been employed to produce composite hydrogels. There are several strategies to produce composite hydrogels, such as physical blending [35,36,37,38], in situ synthesis [39,40,41], bio-conjugation [42,43,44], and forming of interpenetrating polymer networks (IPNs) [45,46,47,48].

In addition to presenting good mechanical properties and response to stimuli such as temperature and/or pH, the hydrogels obtained for biomedical applications must offer a high level of biocompatibility and biomimetics, thus providing biophysical and biochemical properties capable of inducing the correct biological response for both biomedical applications in vitro and in vivo. Finally, they must be biodegradable and preferably have anti-inflammatory and antibacterial properties [49,50].

In this review, we discuss different types of hybrid hydrogels and composites based on PNVCL, as well as their potential use in the biomedical field over the last ten years. We briefly highlight the importance of PNVCL, the most commonly used methods for obtaining hydrogels, general characteristics of PNVCL hydrogels, copolymers and filler materials that are used to obtain hybrid and composite hydrogels, respectively, as well as the promising properties for its application as biomaterials.

**Figure 1:**
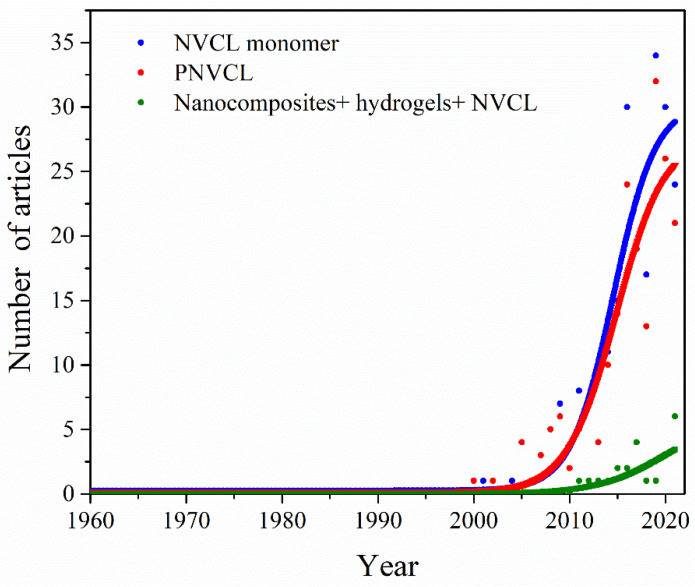
Curve indicating an increase in publications associated with the keywords: *N*-vinylcaprolactam, poly(*N*-vinylcaprolactam) and composites + hydrogel + *N*-vinylcaprolactam during the past 62 years.

The search for *N*-vinylcaprolactam (NVCL), poly(*N*-vinylcaprolactam) and composites + hydrogel + *N*-vinylcaprolactam in the PubMed database shows a low number of articles related to these keywords. Nevertheless, there is a growing trend in the number of published articles, especially from 2000 onwards, as shown in Figure 1.

## 2. Thermo-Sensitive NVCL-Based Hydrogels

### 2.1. Poly(N-vinylcaprolactam)

Poly(*N*-vinylcaprolactam) (PNVCL) is a temperature-sensitive polymer, with a Lower Critical Solution Temperature (LCST) in water of around 33 °C. This temperature indicates a sudden change in the solvated state; lineal and/or branched polymers based on PNVCL tend to precipitate, while crosslinked polymers go from a hydrated to a dehydrated state. An important aspect of this phenomenon is that the LCST can be precisely tuned up and down by incorporating increasing amounts of *N*-vinylacetamides and vinylesters, respectively. Studies have shown adjustments of LCST from 33 °C to 80 °C, with the addition of *N*-vinylpyrrolidone [51] or *N*-vinyl-*N*-acetamide [52]. In addition, PNVCL has solubility in water and organic solvents, high absorption ability, possess a classical Flory–Huggins thermoresponsive phase diagram, and the phase transition depends on the molecular weight and on the solution concentration [53,54].

**Figure 2:**
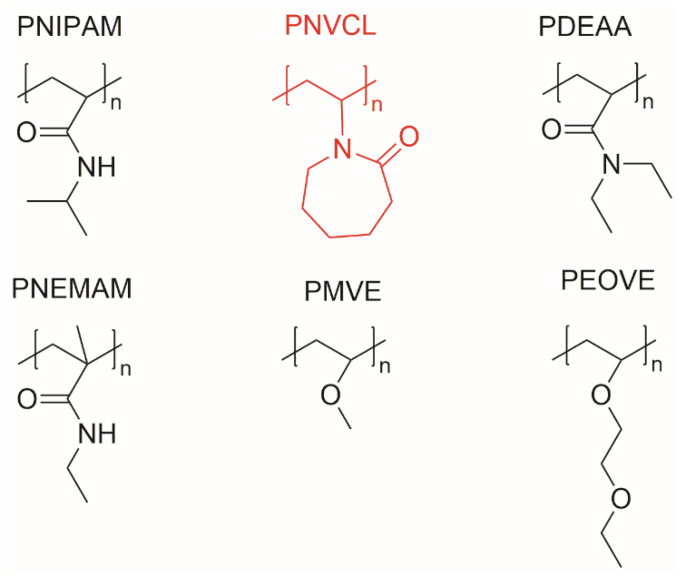
Chemical structures of some thermosensitive polymers.

Although the controlled polymerization and copolymerization for PNVCL are more difficult to achieve [55], compared with other thermosensitive polymers (Figure 2) [8], polymers based on PNVCL have gained great attention in biomedical areas such as: drug delivery systems, antibiotics, tissue engineering and diagnostics and imaging, because of their biocompatible, thermoresponsive and water-soluble behaviour below the LCST [9,56].

In the case of LCST-showing polymers, when they are crosslinked, the concept of LCST changes to a volume-phase transition temperature (T_VPT_) since a volume change (shrinkage) by heating and swelling by cooling is macroscopically observed.

### 2.2. PNVCL-Based Hydrogels by Chemically Crosslinking Methods

**Figure 3:**
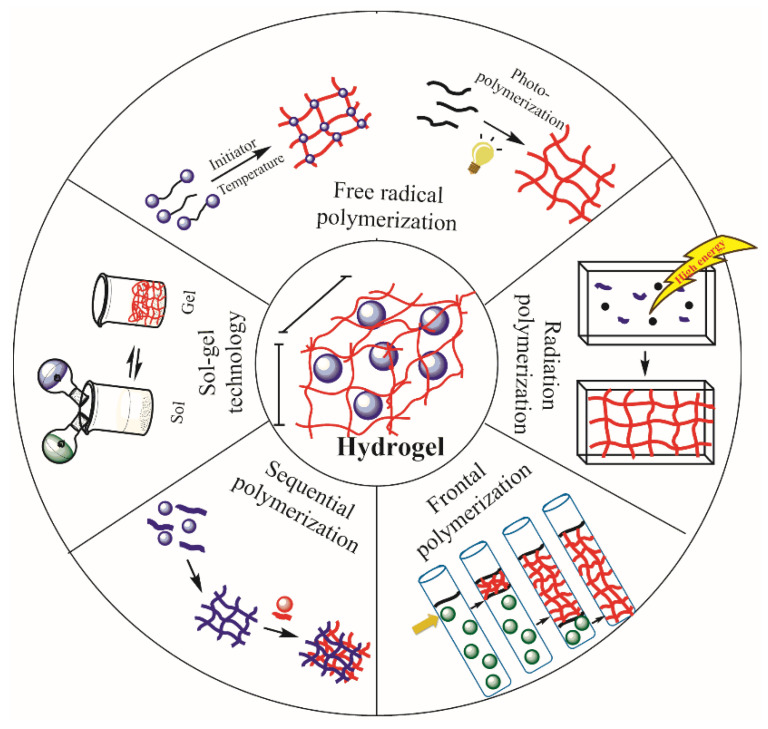
Synthesis of hydrogels by different techniques.

Hydrogels can be synthesized by different techniques. Usually, physical crosslinking, grafting polymerization, radiation crosslinking and chemical crosslinking are used to obtain hydrogels [57]. However, in this work, we focus on chemically crosslinked polymer networks. For this kind of hydrogel, techniques such as free-radical polymerization, photopolymerization, radiation polymerization, frontal polymerization, and sequential polymerization, have been used. Sol–gel techniques are used in the area of preparation of physically crosslinked structures (Figure 3 and Table 1).

#### 2.2.1. Free-Radical Cross-Linking Polymerization

Free-radical polymerization and controlled free-radical polymerization involve initiation, propagation and termination steps. In the initiation step, visible or ultraviolet light photoinitiators, and thermal or redox initiators, produce free radicals. After initiation, a free-radical active site is generated and is responsible for adding more monomer units to the growing polymer chain [71]. The crosslinking reaction is a result of a free-radical polymerization adding a doubly functionalized molecule in the recipe, named a crosslinker. Lynch et al. synthesized hydrogels containing PNVCL and methacrylated hyaluronic acid by free-radical polymerization, showing volume-phase transition temperatures (T_VPT_) and gelation temperatures in the ranges of 33–34 °C and 41–45 °C, respectively; the hydrogel was used for tissue engineering and showed a higher collagen production in PNVCL-*g*-HA hydrogels with respect to the control (methacrylated hyaluronic acid, meHA) [58]. In another report, Arellano-Sandoval et al. obtained agave xylan-type hemicellulose functionalized with trimethoxysilylpropyl methacrylate and crosslinked it with *N*-vinylcaprolactam; the hydrogels showed an interconnected and porous architecture with a T_VPT_ close to PNVCL LCST, with a good capacity to load ciprofloxacin and capacity to inhibit growth of some bacteria [59].

##### Photopolymerization

Photo-initiated polymerization is a type of free-radical polymerization. It is carried out by low-energy radiation, usually UV radiation. This technique is easy to operate, cost-effective, involves fast crosslinking rates at room temperatures, produces minimal heat and, importantly, is an environmentally friendly technique [72]. However, by means of this technique, physical hydrogels have mainly been obtained [60,61,62]. Nevertheless, Shi et al. reported the synthesis of chemically crosslinked PNVCL hydrogels using *N*,*N*′-methylene bisacrylamide (BIS). These hydrogels were compared to clay nanocomposite hydrogels (PNVCL-clay) and the last ones showed better mechanical characteristics [63]. Most recently, Cerda-Sumbarda et al. reported the synthesis of PNVCL chemically crosslinked hydrogels using 3,9-divinyl-2,4,8,10 tetra-oxaspiro [5.5] undecane (DVA) as crosslinker and compared it with composite hydrogels, with nanogel or clay as fillers. Composite hydrogels showed better thermal response and mechanical properties compared to PNVCL hydrogels [64]. According to these reports, it can be assumed that the PNVCL hydrogels obtained by photopolymerization have weak mechanical properties, so their study is complemented with composite hydrogels.

#### 2.2.2. Radiation Polymerization

Radiation polymerization involves high-energy radiations such as gamma and electron beams. These radiations can crosslink water-soluble monomers or polymer chains without using a crosslinker [73]. Similar to free-radical polymerization, there are initiation, propagation and termination steps. Hydroxyl radicals are formed when the irradiation is performed in water, while peroxides are generated when the irradiation is performed in air to rapidly trigger chain propagation. The synthesis of NVCL-DMAAm (*N*-dimethylacrylamide) grafted onto chitosan networks was carried out by gamma irradiation using direct and indirect (pre-irradiation oxidative) methods [65]. To evaluate the influences of architecture on the properties of hydrogels, syntheses in one and two steps were proved. The maximum grafting percentages were obtained by the direct method and the products showed a critical pH increase from 3.8 to 5.2 and a T_VPT_ between 34 and 37 °C for binary systems. On the other hand, only one report for the obtention of NVCL hydrogels using electron beam irradiation, was found. Although physical hydrogels were obtained, increasing the irradiation dose from 5 to 50 kGy provided an increase in the molecular weight distribution due to the formation of chain branching or crosslinking [66].

#### 2.2.3. Frontal Polymerization

Frontal polymerization is a technique that allows for obtaining polymers easily and efficiently, in which a self-propagating exothermic reaction wave transforms liquid monomers to fully cured polymers [74]. This technique was discovered by Chechilo et al. in 1972, and since then, interpenetrating polymer networks, polymer-dispersed liquid crystal materials, polymeric nanocomposites and stimuli-sensitive hydrogels have been prepared [18]. Hydrogels from *N*-isopropylacrylamide (NIPAM) and NVCL were prepared [20]. Authors reported that NVCL influences pore size and shape distribution, and the swelling ratio. The obtained materials were compared with others prepared by classical polymerization, noting that those prepared by frontal polymerization have much larger reversibility % if temperature is allowed to oscillate above and below the T_VPT_. On the other hand, Zhou et al. reported in 2013 the synthesis of poly(*N*-methylolacrylamide-*co*-NVCL) (poly(NMA-*co*-NVCL)) hydrogels by infrared laser-ignited frontal polymerization, as a facile and fast synthesis [18]. Results showed that in 10 s of infrared laser irradiation on the top of the reactor, monomers could be quickly transformed to a polymer, along with a continuous moving and stable front. Data obtained suggest that through this technique, it is possible to carry out energetically efficient synthesis of polymers, which allows for the activation of long-distance reactions via remote control, which is of great interest when working with highly toxic and/or chemically dangerous substances.

#### 2.2.4. Sequential Polymerization

This technique is based on the synthesis of artificial polymers with precise sequence structures. This review highlights multicomponent sequential reactions to prepare periodic sequence-controlled polymers with sufficient molecular diversity and complexity, and hybrid copolymerizations that produce hybrid multiblock copolymers [75]. Interpenetrating polymer networks composed of PNVCL and poly(*N*-acryloyl-*N*′-ethylpiperazine) (PAcrNEP) were obtained by sequential polymerization [21]. Results demonstrate that an interpenetrating polymer network (IPN) is a good substrate for growing nanoparticles, and permeation studies show its ability for drug encapsulation and delivery. Moreover, poly(*N*-vinylpyrrolidone) (PVP)-PNVCL multilayer hydrogels were synthesized [67]. Authors report the effects of hydrophilicity and architecture on the temperature-responsive behaviour and surface morphology of nonionic double-stack hydrogels obtained from crosslinked hydrogen-bonded layer-by-layer films. The hydration of hydrogels consistently increases with the PVP concentration within the network, resulting in suppressed temperature response. In 2017, Zavgorodnya et al. reported nanothin temperature-responsive hydrogels films of PNVCL nanoparticles with high loading capacity for topical drug delivery by layer-by-layer hydrogen-bonded assembly of core–shell PNVCL-*co*-acrylic acid nanoparticles with linear PVP, followed by crosslinking of the acrylic acid shell with either ethylene diamine (EDA) or adipic acid dihydrazide (AAD) [68]. Hydrogels were loaded with sodium diclofenac and studies showed sustained permeation of this drug through an artificial skin membrane as a function of temperature. In 2019, Durkt et al. developed a thermosensitive PNVCL-*g*-aminated alginate (Alg-NH_2_) with a temperature-dependent phase transition close to physiological temperature [69]. Chemical and physical methods were used. Bovine serum albumin was proved for loading and release from hydrogels. In addition, in vitro, cytotoxicity and hemocompatibility analyses were performed and the results confirmed that PNVCL-*g*-Alg-NH_2_ scaffolds are basically non-cytotoxic and non-haemolytic.

#### 2.2.5. Sol–Gel Technology

This technique basically consists of physical interactions between the components of the reaction. The components usually are polymer precursors obtained via free-radical polymerization, which can change from a solution state to a gel state and vice versa when subjected to an external stimulus, such as temperature. There are not many reports on this technique, and in general, the hydrogels obtained are physical hydrogels. In 2017, Sala et al. reported the synthesis of PNVCL hydrogels by this technology [70]. Injectable hydrogels were obtained. Changes in the PNVCL molecular weight and concentration enabled the development of hydrogels with tuneable mechanical properties and fast gelation times (less than 60 s when the temperature was raised from room temperature to physiologic temperature).

## 3. Nanomaterials Used in Composite PNVCL Hydrogels

Since its pioneering work in the 1960s, hydrogel research has shifted from relatively simple single-polymer networks to multifunctional composite hydrogels that better mimic the complex nature of living tissues [70]. The incorporation of nanoparticles to yield composite hydrogels has gained substantial momentum over the years, since these afford tailor-making and extending of material mechanical properties far beyond those achievable through molecular design of the network component. Specifically, the mechanical properties of composite hydrogels are significantly influenced by the shape, orientation, size, continuity, and composition ratios of their reinforcing phase(s). The shapes of the reinforcing elements generally represent particles or fibres, and the orientation of these phases may be random or uniform [49,76,77]. Furthermore, by modifying the polymer chains with stimuli-responsive functional groups, the hydrogel properties can be switched by stimuli including pH, light, magnetic fields, chemical agents, and temperature [1,78,79,80,81,82].

**Figure 4:**
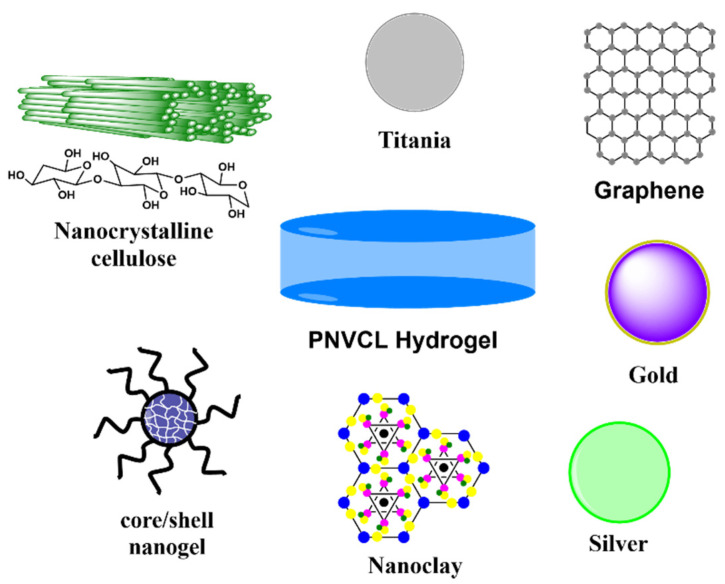
Types of fillers used for the preparation of PNVCL composites hydrogels in the last decade.

Thermoresponsive polymers are those smart polymers which exhibit volume-phase transition, and in turn a sudden solvation state changes at a certain temperature called LCST or UCST. Thermo-responsive polymers, which become insoluble upon heating, display a lower critical solution temperature (LCST) property, while some which are soluble upon heating have an upper critical solution temperature (UCST) property [83,84,85,86,87,88]. In fact, it is this feature that makes LCST-type polymers attractive as smart tools in material and biomedical sciences. Based on a variable temperature input, shrinking/swelling (for crosslinked polymers) or aggregation/dispersion of polymer units (linear or grafted polymers) leads to controllable microscopic or macroscopic changes [69,89,90]. PNVCL is a water-soluble, non-ionic, non-adhesive, non-toxic, biocompatible polymers with an LCST of approximately 31–34 °C in aqueous medium. The cyclic structure of caprolactam ensures the amphiphilic nature of PNVCL, and it does not produce toxic compounds when degraded [91,92,93,94]. In spite of its many advantages of PNVCL, its study as a matrix for the incorporation of nanoparticles is limited since few reports have been generated in the last decade, which are summarized in Table 2. Most of the current reports of PNVCL used micro/nanogels [9,95,96,97]. Therefore, this review focuses on the studies generated in the last decade regarding macroscopic PNVCL hydrogels filled with different nanomaterials (Figure 4).

In 2012, the synthesis of graphene-filled PNVCL hydrogels by a frontal polymerization technique was reported. The nanocomposite polymer hydrogels of PNVCL containing graphene were prepared by varying the amount of the nanofiller from 0.0088 to 0.44 wt%. These materials obtained with graphene nanocomposites have an influence on the swelling ratio (SR%); namely, SR% increases from 1700% for the neat polymer to 2400% for the nanocomposite with the lowest graphene content (0.0088 wt%). Such an SR% increase is a clear indication that graphene largely interacts with the polymer matrix, affecting its typical properties even if present in small quantities. SR% increases with increasing graphene content and reaches a maximum value (3300%) for the hydrogel containing 0.088 wt% graphene. Furthermore, the increase in SR% as graphene increases may be attributed to the disturbance of this nanoparticle on the crosslinking occurrence. The nanofiller does not affects the T_VPT_, since it is always located at ca. 32–33 °C, a value that is close to the physiological temperature. A study on the rheological properties evidenced that the storage modulus (G′) and complex viscosity of the hydrogels decreases as the amount of nanofiller increases, thus indicating that graphene exerts a lubrication effect [19]. Furthermore, Sanna et al. published in 2013 the synthesis of nanocomposite polymer hydrogels of PNVCL containing different amounts of nanocrystalline cellulose (CNC), which are included between 0.20 and 2.0 wt% of the monomer. The introduction of nanocrystalline cellulose involves a strong increase in the hydrophobic character of the polymer, leading to its sharp contraction. Moreover, CNC can act as a physical crosslinker, giving rise to more junctions in the hydrogel network and thus increasing the crosslink density. Therefore, when performing the swelling behaviour, SR% from 1200% was reported for the neat polymer, and with the enhancement of the CNC amount, SR% decreases and reaches the minimum value of 870% for the hydrogel containing 1.0 wt% of CNC. The introduction of CNC in PNVCL hydrogels does not influence the T_VPT_, located around 33–34 °C, a temperature that is very close to that of the human body. The results of rheological analysis in terms of storage (G′) and loss (G″), reported G′ is always higher than G′′ for neat PNVCL and all nanocomposites in the whole frequency range, thus indicating that the material response is prevalently elastic. As expected, nanocomposites have higher moduli than pure PNVCL, which increase with the CNC concentration. The increase in G′ confirms that cellulose nanocrystals act as reinforcement of PNVCL hydrogels, improving their stiffness [98].

Another approach to fillers in hydrogels is noble metal nanoparticles, which are attractive due to their uniqueness, such as resistance to corrosion and oxidation, and non-reactiveness. Among the noble metals NPs, Au and Ag NPs are the most commonly studied nanomaterials. The interesting properties of these noble metal NPs are their high surface-to-volume ratio, wide optical properties, ease of synthesis, and facile surface chemistry and functionalization. Along with improving the physical and chemical properties of the hydrogel, most of the metal NPs are bioactive and naturally possess antibacterial, anti-viral, and anti-inflammatory actions [99,100,101].

In this sense, three studies on PNVCL hydrogel composites with metallic nanoparticles were reported in 2014. The dual responsive pectin hydrogels from poly(acrylamidoglycolic acid-*co*-vinylcaprolactam)/Pectin (PAV-PC) are used as templates for the production of silver nanoparticles. This hydrogel is biodegradable and shows apparent temperature and pH responsiveness. The silver nanoparticles are formed not only on the surface of PAV-PC hydrogels but also throughout the hydrogel networks. In addition, the equilibrium swelling ratio of PAV-PC silver nanocomposite hydrogels is slightly higher than their PAV-PC hydrogels due to their internal network structure [102]. Similarly, sodium alginate and poly(acrylamide-*co*-*N*-vinylcaprolactam-*co*-acrylamidoglycolic acid)-based dual-responsive semi-IPN hydrogels sodium alginate-poly(acrylamide-*co*-*N*-vinylcaprolactam-*co*-acrylamidoglycolic acid) (SA-PAVA) were successfully synthesized by free-radical redox polymerization. *N*,*N*′-Methylene-bis-acrylamide was used as a crosslinker. The hydrogels were also used as templates for the production of silver nanoparticles by using NaBH_4_ as a reducing agent. The thermal stability of hydrogels when combining sodium alginate and all functional monomers (neat SA-PAVA hydrogel and SA-PAVA-Ag) was investigated. A clear difference has been observed in thermogravimetric analysis (TGA) experiments in that SA-PAVA-Ag hydrogel exhibits better thermal stability than the neat hydrogel. Furthermore, the results indicate that the silver nanoparticles were released for a long time when exposed to external stimuli. This is due to the fact that hydrogels contain many hydrophilic functional groups, which are responsible for decreasing the interaction between silver nanoparticles owing to more favourable interactions between hydrogel chains. Thus, the silver nanoparticles are more stable in hydrogel networks. These hydrogels might be effectively useful for silver-releasing wound dressing applications [103].

In the study reported by Zavgorodnya et al., hydrogels were produced by glutaraldehyde-assisted cross-linking of hydrogen-bonded multilayers of poly(*N*-vinylcaprolactam-*co*-aminopropyl methacrylamide) and poly(methacrylic acid). A layer of glutathione-stabilized gold nanoparticles was introduced within the PNVCL hydrogel to initiate an optical response in the presence of anions. When the temperature reversibly changed from 20 °C to 50 °C, the signal intensity of PNVCL-Au hydrogels and the plasmon band position were remarkably dependent on ion concentration and type. These results are essential to understanding the effect of Hofmeister anions on ultrathin non-ionic polymer networks. In addition, a distinct and fast optical monitoring of hydrogel temperature-triggered response depending on ion concentrations can be possible for the PNVCL-Au hybrid hydrogels [104].

Another study shows layered silicates that have been incorporated into hydrogels to enhance their mechanical strength, recoverability, and biological cues by providing physical crosslinking, which is beneficial for the scaffolds, drug carrier or any kind of bio-substrates. Clay/polymer nanocomposite hydrogels can be activated as well as controlled by various external stimuli including temperature, pH, magnetic fields, light, and electric fields [105,106]. Shi et al. [63] showed that due to the nanocomposite structure, the hydrogel exhibits better mechanical characteristics in comparison to the conventional PNVCL hydrogels cross-linked by *N*,*N*′-methylene diacrylamide (BIS). The prepared PNVCL–Clay hydrogel possesses remarkable temperature-responsive characteristics with a volume-phase transition temperature (T_VPT_) around 35 °C, and provides a feasible platform for cell culture. With a macroporous structure and good mechanical characteristics, as well as flexible assembly performance, the proposed biocompatible, thermoresponsive PNVCL–Clay nanocomposite hydrogels are ideal material candidates for biomedical, analytical, and other applications, such as entrapment of enzymes, cell culture, tissue engineering, and affinity and displacement chromatography. Parameswaran-Thankam et al. [107] reported the preparation of a hydroxypropyl guar–*grafted*-PNVCL (HPG-*g*-PNVCL) copolymer modified with nano-hydroxyapatite (n-HA) by in situ covalent cross-linking using a divinyl sulfone (DVS) cross-linker to obtain an HPG-*g*-PNVCL/n-HA/DVS composite material. The scaffold showed apatite-like structure formation on the surface after soaking it for 7 days in simulated body fluid (SBF) solution. As the immersion period was increased up to 14 days, the Ca/P ratio also increased. Therefore, this novel n-HA-containing biocompatible and plant-based hydrogel has promising potential applications in bone tissue regeneration because of its calcium-rich, apatite-forming ability and good bioactivity. Cerda-Sumbarda et al. [64] prepared PNVCL hydrogels by radical photopolymerization with nanoclay charge up to 1 wt%, adding a chemical crosslinker. The PNVCL hydrogels filled with 1 wt% of nanoclay and 6 mol% of crosslinking show better thermal and mechanical behaviour at 25 °C, increasing the Young’s modulus up to 128% compared with the hydrogel without filler. Furthermore, both PNVCL hydrogels with and without fillers showed a phase transition temperature response at temperatures between 32 and 37 °C, the nanoclay filler shifted the transition temperature to higher values. At 37 °C, the nanocomposite hydrogels shrunk. The obtained nanocomposite PNVCL hydrogels have great potential as responsive biomaterials with compression moduli in the megapascal regime.

Titania is a well-studied photoactive compound with excellent mechanical, optical, and physical properties. Furthermore, TiO_2_ possesses high chemical stability, superhydrophilicity, biocompatibility, and reusability, and can be produced at low cost. TiO_2_ is a semiconductor and has a band gap of 3.2 eV (λ < 390 nm). It exists in an amorphous phase that is non-photoactive. Furthermore, there are three natural crystalline modifications: anatase, rutile, and brookite. Given the fact that TiO_2_ has such outstanding properties, it is widely used in various (hybrid) materials [108]. However, our discussion is restricted to the studies of PNVCL hydrogels filled with titania nanoparticles (TiO_2_), which are limited.

Timeva et al. [109] synthesized PNVCL hydrogels with nanosized anatase (A particles) by the sol–gel method, and characterized them in different states by powder X-ray diffraction and scanning electron microscopy. The nanosized anatase particles exert a significant effect on the water content of the hydrogels and on the microstructure of A/PNVCL. The surface of the A/PNVCL composite hydrogel is more developed, and its internal structure comprises pores of smaller sizes compared with neat PNVCL hydrogel, which can be attributed to higher water concentrations in the pores. The water swelling capacity of A/PNVCL is higher than that of the initial PNVCL. The same group of authors reported in 2020 on the morphologies and dynamics of PNVCL-based hydrogels with titania nanoparticles in different states (native, air-dried to a constant weight and swollen in H_2_O or D_2_O), as studied by a combination of complementary techniques: wide-angle X-ray scattering, small-angle neutron scattering, neutron spin echo spectroscopy, and differential scanning calorimetry. PNVCL-based hydrogels with titania nanoparticles were synthesized by admixing TiO_2_ nanoparticles with PNVCL and tetraethoxysilane (TEOS). Titania nanoparticles (NT) and PNVCL are bonded via hydrogen bonding between -N-C=O group and water molecules and OH-groups on the surface of NT. Titania nanoparticles do not change the equilibrium swelling degree of PNVCL-based hydrogels but affect their response rate to the temperature jump 20 → 50 °C. Moreover, the NT concentration changes the form of the response curve [110].

**Table 2:** Recent studies in the field of composite PNVCL hydrogels.

Composite Hydrogel	Nanoparticles Type	Effects	T_VPT_	Reference
PNVCL/Graphene	Inorganic	- Graphene influences the swelling ratio of the obtained hydrogels. - Graphene exerts a lubrication effect.	32–33 °C	[19]
PNVCL/CNC	Polymeric	- The viscosity increases with CNC content, indicating that the nanocellulose has a great potential to reinforce PNVCL polymer hydrogels.	33–34 °C	[101]
PAV-PC/Ag	Metallic	- The equilibrium swelling ratio of PAV-PC silver nanocomposite hydrogels are slightly higher than their PAV-PC hydrogels, due to their internal network structure.	---	[102]
SA-PAVA/Ag	Metallic	- The SA-PAVA-Ag hydrogel exhibits better thermal stability than the neat hydrogel. - The silver nanoparticles are more stable in hydrogel networks.	30 °C	[103]
(PNVCL)_81_/Au	Metallic	- Demonstrated the potential for tracking temperature-induced changes in hydrogels in electrolyte solutions using optical markers.	20–50 °C	[104]
PNVCL/NanoClay	Inorganic	- Macroporous structure and good mechanical characteristics as well as flexible assembly performance, and biocompatibility.	35 °C	[63]
HPG-*g*-PNVCL/n-HA/DVS	Inorganic	- The scaffold showed apatite-like structure formation on the surface after soaking for 7 days in SBF solution. - The Ca/P ratio increased.	34 °C	[107]
A/PNVCL	Inorganic	- The A/PNVCL hydrogel exhibits a twice as high swelling capacity in water as that of PNVCL.	---	[109]
PNVCL/Titania	Inorganic	- The presence of nanoscale titania does affect the microstructure, changing the response rate to a temperature jump from 20 to 50 °C. - Titania nanoparticles do not change the equilibrium swelling degree of hydrogels.	20–50 °C	[110]
PNVCL/Nanogel PNVCL/Nanoclay	Polymeric Inorganic	- Young’s moduli of swollen hydrogels increase up to 278% by using nanogels fillers (0.3 wt%)	32 °C 37 °C	[64]

Nanogels are materials used as fillers in hydrogels since they impart a larger swelling capacity, a higher thermal stability and improved mechanical properties to the hydrogels [33]. In one study, synthesized PNVCL hydrogels were filled with nanogels as soft fillers. The nanocomposite hydrogels were prepared by radical photopolymerization with nanoparticle loads of up to 1 wt%, adding a chemical crosslinker. PNVCL hydrogels filled with 190 nm nanogels are thermally and mechanically improved materials, since the shell of these nanogels is prone to interactions with the carbonyl of the PNVCL and form entanglements, increasing the Young´s modulus up to 278% compared with the hydrogel without filler. Nanocomposite hydrogels show potential application as responsive biomaterials [64].

## 4. Biomedical Applications of PNVCL-Based Hydrogels

**Figure 5:**
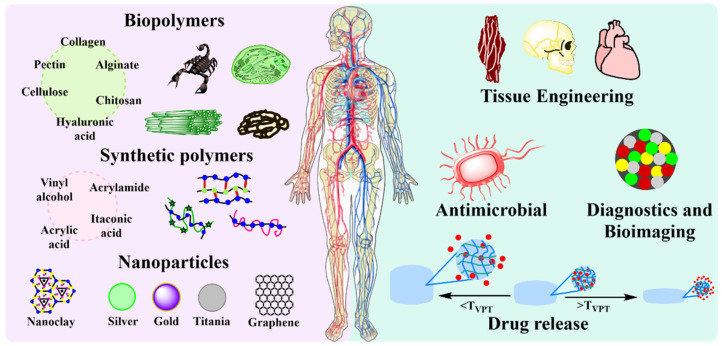
Schematic representation of the biomedical applications of PNVCL hydrogels in the last decade.

The generation of polymeric biomaterials with improved mechanical properties and response capacity, as well as presenting biocompatibility, is the new focus in scientific research. Recently, PNVCL is one of the most used polymers for the development of materials with biomedical applications due to its ability to respond to stimuli such as temperature and its proven biocompatibility. The thermal responsiveness of PNVCL, used alone or in combination with other stimuli-responsive polymers or particles (pH, magnetic field, or chemicals), is often key for the attractive different applications [9,110,111] which includes drug delivery systems [5,69,81,101,106,112,113,114], antibiotics [59,102,107], tissue engineering [58,88,106], and diagnostics and imaging [115,116]. Figure 5 outlines studies in recent years on PNVCL for biomedical applications.

### 4.1. Antibacterial and Drug Delivery Systems

In traditional drug administration, high doses or repeated administration are generally used to increase the therapeutic effect, however, this can cause a reduction in the general efficacy of the drug and generate side effects and even systemic toxicity, decreasing the quality of life of the patient. Hydrogels offer convenient drug delivery vehicles to ensure these disadvantages are minimized and provide spatial and temporal control over the release of various therapeutic agents, including small-molecule drugs, macromolecular drugs and cells. Owing to their tuneable physical properties, controllable degradability and capability to protect labile drugs from degradation, hydrogels emerge as very efficient drug delivery systems [112,113,114,117].

PNVCL is one of the most important thermoresponsive polymers because it shows reversible thermoresponsive behaviour in a physiological temperature range (32–34 °C). The use of PNVCL is considered advantageous because of its lower toxicity compared with other thermoresponsive polymers such as PNIPAM. Hybrid PNVCL hydrogels are sensitive to external stimuli, such as temperature and pH, which gives them a wide range of biomedical applications and consequently attracts considerable scientific interest [8]. Table 3 outlines the recent significant utilization of PNVCL in antimicrobial and drug-release applications.

The incorporation of cationic and anionic monomers further showed double (pH and thermo) responsive behaviour of the PNVCL hydrogels [111]. Novel dual-responsive PAV-PC hydrogels are used as templates for the production of silver nanoparticles. In addition, 5-Fluorouracil release from poly(acrylamidoglycolic acid-*co*-*N*-vinylcaprolactam)-pectin (PAV-PC) hydrogels was 50% at pH 1.2 and 85% at pH 7.4 within 24 h. The release profile with PAV-PC hydrogels showed that an initial burst effect was significantly reduced in two buffer media (1.2 and 7.4), followed by a continuous and controlled release phase; the drug release mechanism from the polymer was due to Fickian diffusion. In vitro antimicrobial activity assessments exhibited that PAV-PC silver nanocomposites showed a pronounced inhibitory effect against the two bacteria strains (*B. subtilis* and *E. coli*) [101] The same authors synthesized dual-responsive semi-IPN hydrogels (SA-PAVA) and studied them as a drug delivery system. In addition, 5-FU, as a model anticancer agent, was used for encapsulation into these semi-IPNs via an equilibrium swelling method. In addition, the authors synthesized silver nanoparticles (Ag NPs) and proved that the gels could control the release of the drug and Ag NPs through stimuli such as pH and temperature [102].

Indulekha et al. designed a heat-triggered transdermal drug delivery system (TDDS) using a thermoresponsive polymer, a PNVCL-based gel, where patients can themselves administer a pulse of drug on mere application of heat pad over the TDDS, whenever pain is experienced. Chitosan-*g*-PNVCL (CP) hydrogels were subjected to comparative studies on loading, in vitro temperature and pH-dependent triggered release and temperature-dependent skin permeation of acetamidophenol and etoricoxib drugs. Liquid chromatography–mass spectrometry (LC-MS) analyses on the permeated drugs showed the triggered response of the heat in the skin, and the transdermal CP gel formulation had a significant role in drug permeation through skin in vitro. The hydrophobic drug showed better in vitro skin permeation in rat abdominal skin than that of the hydrophilic drug. Histopathological results proved that the CP gel was found to be biocompatible by the skin irritation test performed in rat skin in vivo. Thus, the drug-loaded CP gel formulation could be a potential candidate for an on-demand transdermal drug delivery system for pain management [112].

Durkut et al. investigated the release of model drugs—bovine serum albumin and lidocaine hydrochloride—from a poly(*N*-vinylcaprolactam)-*g*-collagen (PNVCL-*g*-Col) sponge as a function of time. Release patterns are similar, observing a rapid release in the first couple of hours and then reaching an asymptote at around 72  h and 48  h for bovine serum albumin (BSA) and lidocaine, respectively. Cumulative release at 4  °C was higher than at 40  °C at all times. This can be explained by the swollen state of PNVCL-*g*-Col below the T_VPT_ (4  °C), allowing drug release. Due to the collapse of the hydrogel network, percent cumulative release above the T_VPT_ (40  °C) was −40% lower for BSA, and 30% lower for lidocaine [118].

On the other hand, Ciprofloxacin, a commonly known antibiotic, was used for sustainable release from the hydroxypropyl guar-*graft*-poly(*N*-vinylcaprolactam) (HPG-*g*-PNVCL) hydrogel as a function of time at 37 °C because of its viscous nature and thermogelation of the copolymer. Ciprofloxacin release from 3 wt% HPG-*g*-PNVCL, 4 wt% HPG-*g*-PNVCL, and 5 wt% HPG-*g*-PNVCL hydrogels showed a biphasic manner with initial burst release, followed by a controlled release pattern of sustained release. For instance, the release is slower for formulations containing a high concentration of HPG-*g*-PNVCL. This may be due to the presence of higher grafted polymers which undergo chain entanglements among themselves, leading to the entrapment of drug molecules tightly and them being released in a controlled manner [106].

In 2019, a dual (thermo- and pH-)-responsive hybrid copolymer, poly(*N*-vinylcaprolactam)-*grafted*-galactosylated chitosan (PNVCL-*g*-GC), was synthesized by carbodiimide [*N*-(3-dimethylaminopropyl)-*N*′-ethylcarbodiimide]/*N*-hydroxysuccinimide (EDC/NHS) crosslinking. PNVCL-*g*-GC hydrogel was evaluated as a delivery vehicle dependent on environmental pH and temperature. A faster bovine serum albumin release was observed at 40  °C and at pH 7.2 from the cytocompatible and hemocompatible copolymer [119]. The same authors developed thermosensitive poly(*N*-vinylcaprolactam)-*grafted*-ammino alginate (PNVCL-*g*-Alg-NH_2_) hydrogels with a temperature-dependent phase transition close to physiological temperatures. In the study of a model protein (BSA), the release from PNVCL-*g*-Alg-NH_2_ scaffolds indicates a higher rate of release below the T_VPT_ than above it, owing to the fact that PNVCL chains collapse above the T_VPT_ and form a more compact network. In vitro cytotoxicity and hemocompatibility analyses confirm that PNVCL-*g*-Alg-NH_2_ scaffolds are basically non-cytotoxic and non-haemolytic [67]. Fallon et al. synthesized hydrogels with dual sensitivity, and acetaminophen release studies were carried out to establish the pH sensitivity of the hydrogels. The incorporation of itaconic acid (IA) allowed for an increase in the acetaminophen released at pH 6.8, while at pH 2.2, small amounts of the drug were released. Poly(*N*-vinylcaprolactam-*co*-itaconic acid) hydrogels may have the potential to protect a drug in the acidic conditions of the stomach and release a drug in response to changes in the physiological pH, making these materials very attractive for targeted drug delivery. The hydrogels prove to be highly biocompatible and show no evidence of toxic side effects. Overall, these hydrogels have potential use as an oral drug delivery system [81].

A novel temperature- and pH-responsive hydrogel was prepared by combining PNVCL and hydrolysed epoxy soybean oil-grafted hydroxyethyl cellulose (H-ESO-HEC). The controlled-release experiment showed that both pH change and temperature change significantly accelerated the release of salicylaldehyde. Based on these results, those dual-responsive hydrogels have the potential application for a controlled release of drugs [5]. Another strategy for drug release tested was the synthesis of a thermo- and pH-sensitive binary graft, based on NVCL and pH-sensitive acrylic acid (AAc) monomers, onto chitosan gels (*net*-CS) via ionizing radiation. Preliminary studies of drug retention were not entirely successful because the sizes of the pores were too small for the diffusion of molecules as large as those of vancomycin. However, diclofenac had better retention because of its small size and negative charge. The load and release of diclofenac showed that the grafted system (32%) was able to load 19.3 mg/g and release about 95% at 200 min in comparison to crosslinked chitosan, which only released 80% in the same period. This was possibly caused by the UCST-driven response behaviour, which allows the pores of the grafted system to be open at physiological temperature (37 °C). Moreover, when the grafted sample was protonated, the loaded drug was higher (27 mg/g), but only 20% was released because of the strong electrostatic interactions between the sample and diclofenac [120].

In a recent work, a first temperature-responsive hydrogel was successfully produced from a plant’s natural polymer (epoxidized natural rubber) and PNVCL/poly(vinyl alcohol) blend for the first time by a graft reaction using potassium persulfate (KPS) as an initiator. A sustained release profile of curcumin from this matrix was found and the release percentage of the loaded curcumin sample at 40 °C and 72 h was much lower than the release percentage of the sample at 25 °C [121].

On the other hand, infectious diseases caused by pathogens remain a major health threat, even though there have been major advancements in the standards of the medical technology and healthcare fields [26,68]. Recently, the development of PNVCL hydrogels has become an attractive strategy, however, in recent years there have been few studies on this topic. In 2014, two dual-response formulations were developed using silver nanocomposites in PNVCL hydrogels combined with pectin [101] and alginate [102]. Both systems showed a pronounced inhibitory effect against the two bacteria (*B. subtilis* and *E. coli*) tested in vitro in evaluations of antimicrobial activity. Arellano-Sandoval et al. successfully prepared hybrid hydrogels from silanized hemicellulose (HC) and PNVCL by chemical crosslinking through radical polymerization between PNVCL and silanized HC. These materials showed a good capacity to load ciprofloxacin (in the range 9.5 × 10^−3^–8.4 × 10^−3^ mg/mL), above the minimum inhibitory concentration (MIC ≤ 0.004 × 10^−3^–0.5 × 10^−3^ mg/mL) for Gram-positive and Gram-negative bacteria, and apparently the same capability permitted the controlled release of the antibiotic in time. This effect of inhibition mediated by the composite’s characteristic release of the antibiotic in “stages” confers the ability to inhibit the growing of the bacteria strains (*Escherichia coli, Staphylococcus aureus*, and *Pseudomonas aeruginosa*) for at least 3 days, making this a promising formulation to apply in surgical wounds, where a desirable characteristic is presented by hybrid hydrogels [59].

### 4.2. Tissue Engineering, Diagnostics and Bioimaging

Tissue engineering is an evolving and interdisciplinary field that has the potential to provide permanent solutions to tissue damage and tissue loss to millions of people each year. The basic approach to tissue engineering involves the use of cells, scaffolds, and signalling factors, alone or in combination. The goal of tissue engineering is to provide biological substitutes that can maintain, restore, or improve the function of damaged tissues [122]. In this context, there has been a growing interest among researchers in polymeric hydrogel systems as candidates for developing suitable artificial extracellular matrices (ECMs). For example, in tissue regeneration [106], a given technique must produce a composite that is biocompatible (chemical composition), able to withstand prolonged applications of specific mechanical forces (macrostructure–microstructure), allowing for directed cellular attachment (cell adhesivity), migration (porosity), as well as achieving a conformational architecture with the site of repair. Each key element is necessary to guide the repair and integration of new tissue. Furthermore, these attributes encompass the primary advantages of composite hydrogels in comparison to more traditional hydrogels [76,123]. Recently, PNVCL-based hydrogels have become important because of their biocompatibility and temperature-responsive nature, highlighting their potential in the biomedical field [111]. However, there has been little work towards the use of PNVCL in tissue engineering in the last decade, especially when dealing with the investigation of cell–material interactions and tissue formation in vitro and in vivo [69,124,125].

In 2016, the development of two temperature-sensitive hydrogels to support cartilage tissue engineering was investigated. Linch et al. prepared several composite hydrogels containing PNVCL and methacrylated hyaluronic acid (meHA) using free-radical polymerization to produce PNVCL-*g*-HA. After 10 days at 1% oxygen, collagen production on PNVCL-*g*-HA hydrogels was 153 ± 25 μg/mg (20%) and 106 ± 18 μg/mg, showing a 10-fold increase compared to meHA controls. These hydrogels show promise as new materials for cartilage tissue engineering to improve chondrocyte viability and biochemical synthesis of extracellular matrix proteins under hypoxic conditions [58]. Furthermore, a family of regenerated silk fibroin (RSF)-based double-network (DN) hydrogels were fabricated for the first time using a rapid one-pot method. The DN hydrogels combine the rigid covalently crosslinked RSF with the softer PNVCL through strong physical interactions. The formation of these DN hydrogels resulted in an improvement of the water uptake capacity, elasticity and toughness compared to the individual RSF hydrogel. Tuning the stiffness and water uptake capacity of the hydrogels altered the hydrogel’s capacity to support cellular proliferation. Notably, the less rigid RSF/PNVCL hydrogels exhibited the most favourable chondrogenic differentiation properties. This study shows, for the first time, the potential of RSF-based hybrid hydrogels containing a synthetic polymer (PNVCL) to be used in cartilage tissue regeneration [126].

In a similar approach, Sala et al. in 2017 synthesized thermosensitive injectable hydrogels of PNVCL with tuneable mechanical properties and fast response to temperature stimuli, showing great potential for application to cartilage repair. Chondrocytes (CHs) and mesenchymal stem cells were encapsulated in PNVCL hydrogels and exhibited high viability (∼90%), as monitored by Live/Dead staining and Alamar Blue assays. Three-dimensional constructs of CH-laden PNVCL hydrogels supported cartilage-specific extracellular matrix production both in vitro and after subcutaneous injection in nude rats for up to 8 weeks. Moreover, biochemical analyses of constructs demonstrated a time-dependent increase in glycosaminoglycans (GAGs) and collagen, which were significantly augmented in the implants cultured in vivo. Histological analyses also demonstrated regular distribution of synthesized cartilage components, including abundant GAGs and type II collagen. The findings from this study demonstrate thermosensitive PNVCL as a candidate biomaterial to deliver cells for cartilage tissue engineering [70].

In another scenario, PNVCL hydrogels were synthesized by graft polymerization with hydroxypropyl-guar (HPG) and modified with nano-hydroxyapatite by covalent cross-linking to obtain a composite material. In vitro cytotoxicity studies reveals that the HPG-*g*-PNVCL thermogelling polymer works as a biocompatible scaffold for osteoblastic cell growth. The composite hydrogel was used in an in vitro biomineralization study. The scaffold showed apatite-like structure formation on the surface after soaking for 7 days in simulated body fluid solution. As the immersion period was increased up to 14 days, the Ca/P ratio also increased. Therefore, this novel n-HA-containing biocompatible and plant-based hydrogel has promising potential applications in bone tissue regeneration because of its calcium-rich apatite-forming ability and good bioactivity [106].

Multifunctional systems are becoming increasingly important for applications in a wide range of fields including imaging, biosensing, diagnostics and medicine [115]. Bioimaging plays a crucial role in understanding the morphology, structure, metabolism, and functions of cells. Characteristics to be considered for an ideal imaging agent include sensitivity, specificity, efficiency, optimal clearance and response to disease biomarkers to assist in the diagnosis, assessment and treatment of diseases [123]. Hydrogels are being applied to numerous biomedical applications due to their potential tuneable rheological properties, injectability into tissues, encapsulation and release of therapeutics. Despite their promise, it is challenging to assess their properties in vivo. To address this, technologies to evaluate and track hydrogels in vivo with various imaging techniques have been developed in recent years, including hydrogels functionalized with contrast-generating material that can be imaged with methods such as X-ray computed tomography, magnetic resonance imaging, optical imaging, and nuclear imaging systems [127].

In the literature, two recent works were found where PNVCL nanogels with fluorescent materials were used for diagnostic and bioimaging applications [116,117]. However, there are no studies on PNVCL-based composite hydrogels with fluorescent components. Therefore, the development of hydrogels with fluorescent elements based on PNVCL for in vivo imaging is essential.

## 5. Conclusions and Future Perspectives

PNVCL-based hydrogels are biocompatible and temperature-sensitive materials. They have been copolymerized with other polymers to improve their mechanical properties, to adjust the T_VPT_ or to provide sensitivity to another stimulus such as pH. Likewise, in order to improve the mechanical, chemical, physical and/or biological properties, different nanoparticles have been incorporated into the polymeric networks, such as metallic, inorganic, and, in very few cases, polymeric nanoparticles. However, the number of studies on PNVCL-based hydrogels and composites is currently small.

The applications of PNVCL-based hydrogels in the biomedical area are mainly as drug delivery systems, antimicrobial hydrogels and in tissue regeneration. In the case of hydrogels studied as drug delivery systems and as antimicrobial hydrogels, despite the great advances in understanding the physical, chemical, mechanical and biological properties, depending on the polymerization parameters, there are still many challenges from a clinical perspective. These materials could help solve current challenges in antimicrobial medicine, including antibiotic resistance, through localized, sustainable release of drugs or by combination with different ingredients which may exhibit a synergistic effect [128]. However, current studies on antimicrobials with PNVCL hydrogels are scarce and have only been limited to loading of model antibiotics and silver nanoparticles, as well as testing them on specific bacteria such as *E. coli* and *S. aureus*. For future applications, it is necessary that the research be directed to testing the systems against multi-resistant bacteria, in addition to combine them with other materials with antimicrobial activity.

PNVCL hydrogels applied to tissue engineering have shown a great potential for application in the regeneration of cartilage and bone tissue in in vitro studies, so it is necessary to carry out research where the scaffolds are evaluated in in vivo studies. There is also a need to develop PNVCL hydrogels with a focus on regeneration of other tissues and organs. In addition, diagnostic and imaging applications are a promising field for PNVCL hydrogels, since there are only few reports in this area for nanometer- and micrometer-scale hydrogels. Furthermore, the clinical applications of PNVCL hydrogels are limited and there are no FDA-approved commercial products yet. We expect to see a great development in research and breakthroughs in clinical application of these PNVCL-based hydrogels and composites in the near future.

## Figures and Tables

**Table 1:** PNVCL hydrogels obtained by different techniques.

Polymerization Technique	Description of Hydrogels	Reference
Free-radical polymerization	-NVCL-*g*-HA (hyaluronic acid)	[58]
	-Silanized hemicellulose and NVCL	[59]
Photopolymerization	-NVCL ^1^	[60]
	-NVCL-AAc (Acrylic acid) ^1^	[61]
	-NVCL-VAc (Vinyl acetate) ^1^	[62]
	-NVCL with BIS as crosslinker	[63]
	-NVCL with DVA as crosslinker	[64]
Radiation polymerization	NVCL-DMAAm grafted onto chitosan by gamma irradiation	[65]
	NVCL hydrogels by e-beam irradiation	[66]
Frontal polymerization	PNIPAM-PNVCL	[20]
	Poly(NMA-*co*-NVCL)	[18]
Sequential polymerization		
	PNVCL and PAcrNEP	[21]
	PVP-PNVCL multilayer	[67]
	PNVCL hydrogel films	[68]
	PNVCL-*g*-Al-NH_2_	[69]
Sol–gel technology		
	PNVCL injectable hydrogels ^1^	[70]

^1^ Physical hydrogels.

**Table 3:** Compendium of the novel studies in recent years on PNVCL hydrogel systems for antimicrobial and drug-release applications.

Type of Hydrogel	Drug Delivery/Antimicrobial	Therapeutic Application/Bactericides	Reference
PAV-PC	5-Fluorouracil Silver nanoparticles	Drug delivery system/*Bacillus subtilis* and *E. coli*	[102]
SA-PAVA	5-Fluorouracil Silver nanoparticles	Drug delivery system/*Bacillus subtilis* and *E. coli*	[103]
Chitosan-*g*-PNVCL	Etoricoxib Acetamidophenol	On-demand transdermal drug delivery system for pain management.	[118]
PNVCL-*g*-Col	Lidocaine hydrochloride and bovine serum albumin	Drug delivery system	[119]
HPG-*g*-PNVCL	Ciprofloxacin	Antimicrobial controlled drug delivery	[107]
PNVCL-*g*-GC	Bovine serum albumin	Drug delivery system	[120]
PNVCL-*g*-Alg-NH_2_	Bovine serum albumin	Drug delivery system	[69]
Poly(*N*-vinylcaprolactam-*co*-itaconic acid)	Acetaminophen	Drug delivery system	[82]
Cellulose-based polymeric emulsifier stabilized PNVCL	Salicylaldehyde	Drug delivery system	[5]
Hybrid hydrogels from silanized hemicellulose and PNVCL	Ciprofloxacin	*E. coli*, *S. aureus* and *P. aeruginosa*	[59]
NVCL/AAc onto *net*-CS	Vancomycin Diclofenac	Drug delivery system	[121]
Epoxidized natural rubber-*g*-PNVCL/poly(vinyl alcohol) blend	Curcumin	Drug delivery system	[122]

## Data Availability

The authors declare that all data and materials supporting the findings of this study are available within the article. The data that support the findings of this study are available from the corresponding author upon reasonable request.

## Abbreviations

AAc: acrylic acid; AAD, adipic acid dihydrazide; Alg-NH_2_, aminated alginate; A particles, anatase particles; BSA, bovine serum albumin; EDC, carbodiimide [*N*-(3-dimethylaminopropyl)-*N*′-ethylcarbodiimide]; CP, chitosan-*g*-PNVCL; CHs, chondrocytes; Col, collagen; DVA, 3,9-divinyl-2,4,8,10 tetra-oxaspiro [5.5] undecane; DVS, divinyl sulfone; DN, double network; EDA, ethylene diamine; ECMs, extracellular matrices; 5-FU, 5-fluorouracile; GC, galactosylated chitosan; GAGs, glycosaminoglycans; HA, hyaluronic acid; H-ESO-HEC, hydrolyzed epoxy soybean oil-grafted hydroxyethyl cellulose; HPG, hydroxypropyl-guar; IPN, interpenetrating polymer network; LC–MS, liquid chromatography-mass spectrometry; G′′, loss modulus; LCST; lower critical solution temperature; meHA, methacrylated hyaluronic acid; BIS, *N*,*N*′-methylene diacrylamide; CNC, nanocrystalline cellulose; n-HA, nano-hydroxyapatite; NPs, nanoparticles; DMAAm, *N*-dimethylacrylamide; NHS, *N*-hydroxysuccinimide; NIPAM, *N*-isopropylacrylamide; NMA, *N*-methylolacrylamide; NVCL, *N*-vinylcaprolactam; PC, pectin; PEOVE, poly(2-ethoxyethyl vinyl ether); PAVA, poly(acrylamide-*co-N*-vinylcaprolactam-*co*-acrylamidoglycolic acid), PAV, poly(acrylamidoglycolic acid-*co*-*N*-vinylcaprolactam); PMVE, poly(methyl vinyl ether); PDEAA, poly(*N*,*N*-diethylacrylamide); PAcrNEP, poly(*N*-acryloyl-*N*′-ethylpiperazine); PNEMAM, poly(*N*-ethylmethacrylamide); PNIPAM, poly(*N*-isopropylacrylamide); PNVCL, poly(*N*-vinylcaprolactam); PVP, poly(*N*-vinylpyrrolidone); PP, polypropylene; KPS, potassium persulfate; RSF, regenerated silk fibroin; semi-IPN, semi interpenetrating polymer network; SR, silicone rubber; SBF, simulated body fluid; SA, sodium alginate; G′, storage modulus; SR%, swelling ratio; HC, silanized hemicellulose; TEOS, tetraethoxysilane; TGA, thermogravimetric analysis; NT, titania nanoparticles; TDDS, transdermal drug delivery system; UCST, upper critical solution temperature; VAc, vinyl acetate; T_VPT,_ volume phase transition temperature.
